# New journal selection for quantitative survey of infectious disease research: application for Asian trend analysis

**DOI:** 10.1186/1471-2288-9-67

**Published:** 2009-10-06

**Authors:** Hiromi Takahashi-Omoe, Katsuhiko Omoe, Nobuhiko Okabe

**Affiliations:** 1 Science and Technology Foresight Center, National Institute of Science and Technology Policy, Ministry of Education, Culture, Sports, Science and Technology, Tokyo, Japan; 2 Department of Veterinary Medicine, Iwate University, Iwate, Japan; 3 Infectious Disease Surveillance Center, National Institute of Infectious Diseases, Tokyo, Japan

## Abstract

**Background:**

Quantitative survey of research articles, as an application of bibliometrics, is an effective tool for grasping overall trends in various medical research fields. This type of survey has been also applied to infectious disease research; however, previous studies were insufficient as they underestimated articles published in non-English or regional journals.

**Methods:**

Using a combination of Scopus™ and PubMed, the databases of scientific literature, and English and non-English keywords directly linked to infectious disease control, we identified international and regional infectious disease journals. In order to ascertain whether the newly selected journals were appropriate to survey a wide range of research articles, we compared the number of original articles and reviews registered in the selected journals to those in the 'Infectious Disease Category' of the Science Citation Index Expanded™ (SCI Infectious Disease Category) during 1998-2006. Subsequently, we applied the newly selected journals to survey the number of original articles and reviews originating from 11 Asian countries during the same period.

**Results:**

One hundred journals, written in English or 7 non-English languages, were newly selected as infectious disease journals. The journals published 14,156 original articles and reviews of Asian origin and 118,158 throughout the world, more than those registered in the SCI Infectious Disease Category (4,621 of Asian origin and 66,518 of the world in the category). In Asian trend analysis of the 100 journals, Japan had the highest percentage of original articles and reviews in the area, and no noticeable increase in articles was revealed during the study period. China, India and Taiwan had relatively large numbers and a high increase rate of original articles among Asian countries. When adjusting the publication of original articles according to the country population and the gross domestic product (GDP), Singapore and Taiwan were the most productive.

**Conclusion:**

A survey of 100 selected journals is more sensitive than the SCI Infectious Disease Category from the viewpoint of avoiding underestimating the number of infectious disease research articles of Asian origin. The survey method is applicable to grasp global trends in disease research, although the method may require further development.

## Background

Quantitative survey of research articles, as an application of bibliometrics, is an effective tool for grasping overall trends and productivity in various medical research fields, such as genetic epidemiology [1], radiological research [2,3], non-communicable disease research [4], tropical medicine [5], public health [6,7], dermatology [8], gastroenterology and hepatology [9], pediatrics research [10], and so on. Additionally, comprehensive analyses of research productivity in the field of biomedical research have been reported [11-14]. The results of analysis contribute to provide the information needed for decision-makers dealing with a specific subject, public policymakers, researchers and business leaders [15]. Also in the field of infectious disease research, quantitative survey to understand study trends on the prevention, detection, diagnosis, and treatment of diseases is an advantage for formulating further research strategies, linked to perspective national and international policies for disease control. Given that there have been frequent outbreaks of various severe emerging infectious diseases, as pandemic (H1N1) 2009 and human cases of H5N1 avian influenza [16,17], such overall analysis of studies has become important.

In the field of infectious disease research, articles about specific infectious diseases, such as Human Immunodeficiency Virus (HIV)/Acquired Immune Deficiency Syndrome (AIDS), have been quantitatively analyzed [18-21]. Meanwhile, there are relatively few comprehensive analyses of all infectious disease research; only several previous studies on the EU [22-24] and specific world regions, including Japan [25-27]. These studies demonstrated the trends on infectious disease research, in viewpoint of the relation to gross domestic product (GDP) [22-24,27], gross national income (GNI) [26], share of research articles [25] and the impact factor (IF) [26], which was developed by Thomson Reuters to quantify citations of scientific journals [28]. However, these studies were limited regarding the collection of research articles as they tended to collect more international English research articles than regional or non-English papers. Articles published in regional journals, particularly Asian journals, should be further analyzed because many outbreaks of emerging infectious diseases, such as severe acute respiratory syndrome (SARS), Nipah virus infection, and human cases of H5N1 avian influenza, have been reported in Asian countries [29] and demand for the control of such diseases might be rising among scientists in these countries. Nonetheless, studies on overall research trends across Asia have not been reported, regardless of specific approaches such as HIV/AIDS research in India [30], tuberculosis research in India and China [31] and Japan's share of articles published in 7 journals, which were considered to have the high IF in the field of infectious disease research [25].

Asian research trends have not been sufficiently analyzed because of limitations regarding journal selection for a survey of research articles. One of the reasons is that several previous analyses [22,24-27] relied on journals registered in the 'Infectious Disease Category' of the Science Citation Index Expanded™ (SCI Infectious Disease Category) [32]. The SCI Infectious Disease Category covers the major journals in the field of the research, however, the vast majority of journals in the category are produced in English [22]. Another reason is that Asian regional journals, particularly in their native languages, tend to be less cited by English-speaking researchers because of the extra effort of translation. This bias possibly disadvantages researchers in Asia, whose study results are published in not only international journals but also their regional journals. Therefore, in order to closely analyze Asian trends in infectious disease research, a new approach with the potential to survey a wide range of journals should be developed.

The objective of this study was to demonstrate a new journal selection applicable to widely survey infectious disease research articles, with less bias among countries and regions. Using these journals, we also experimented with quantitative analysis to understand the trend in infectious disease research in Asia.

## Methods

**Figure 1:**
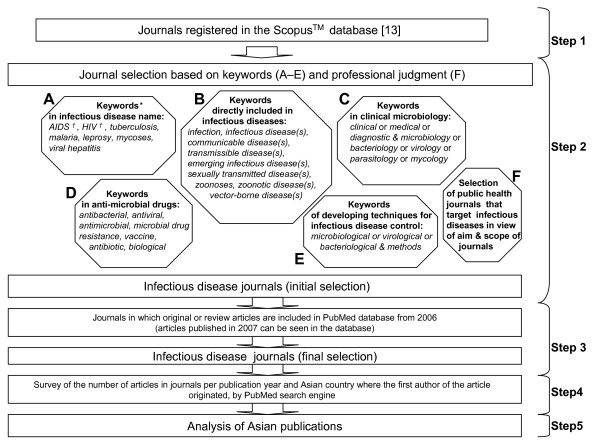
**Framework for quantitative analysis of infectious disease research**. *All keywords in A (except for HIV), infection, infectious disease, communicable disease, sexually transmitted disease, and zoonosis in B, antibacterial, antiviral, antimicrobial, vaccine, and antibiotic in D were translated into French, German, Italian, Spanish, Turkish, Chinese (in Roman letters) and Japanese (in Roman letters) in order to select journals written in non-English languages. ^†^Journals regarding research on AIDS and HIV were selected according to the 'Infectious Disease Category' of the Science Citation Index Expanded™ [32], because several journals specializing in social-scientific and policy studies on patients could not be excluded by only the keywords 'AIDS' and 'HIV'.

The following steps correspond to Figure 1.

### Step 1- Adoption of data source

In this study, we applied the Scopus™ database [33] as a source to select journals on infectious disease research (infectious disease journals). This is a new abstract and citation database of the scientific literature provided by Elsevier B.V., which includes over 16,000 peer-reviewed journals.

### Step 2- Screening of journals

On the basis of the Scopus™ database (registered in January 2008), we screened infectious disease journals, using English keywords directly linked to disease control in detection, prevention, diagnosis and medical care (A-E). The keywords intended to select journals specifically focusing on infectious disease (A), corresponding to general or categorical infectious diseases (B), in the field of clinical microbiology (C), regarding the development of medicine (D), and intended for overall technology development for disease control (E). Additionally, we selected related journals in the field of public health on the basis of our experience (F).

In parallel, we screened non-English journals, using Japanese, Chinese, French, German, Italian, Spanish, and Turkish keywords corresponding to the keywords in A-E (details shown in the legend to Figure 1). To screen Korean journals, we used English keywords, because almost all journal titles (89 of 91 journals) were registered in English, or both English and Korean (in Roman letters) in the Scopus™ database. In the survey with English and non-English keywords, we introduced an approach based on both partial matching (for a search of journal titles that contain the keywords) and complete matching (for a search of titles that perfectly matches the keywords) to capture journal titles involving inflected forms of the keywords.

### Step 3- Selection of journals

After screening the journals in *Step 2*, we finally selected journals in which original or review articles were included in the PubMed database provided by the U.S. National Library of Medicine [34] from 2006 (articles published in 2007 can be seen in the database) because we emphasized the further usability of our survey method: the PubMed database is freely accessed and widely used, and the selected journals have continued in print.

### Step-4 Survey of Asian research articles

For quantitative analysis of infectious disease research articles of Asian origin, we surveyed the number of research articles in the above selected journals that were produced in 11 Asian countries: Japan, China, India, Taiwan, Korea, Singapore, Malaysia, Indonesia, Vietnam, Thailand, and the Philippines. We also surveyed the number of the world total as a reference.

We took advantage of the 'Limits' function of the PubMed database, which contains tags for limiting the journal name (*[Jour]*), affiliation of author (*[ad]*), publication date (*[PPDAT] *for print date and *[EPDAT] *for electronic publication date) and publication type (*[pt]*) [35]. We targeted original articles and reviews as research articles (hereafter, research articles mean original articles and reviews); the former was considered as an indicator of research activity and the latter as an appreciation of research results. Since we considered that highly valued scientists were given more opportunities to write reviews, meaning that their research results had attracted a good opinion and had relatively good qualities, we targeted not only original research articles, but also reviews. Based on the thought that the number of reviews might be indicative of research quality, it was surveyed separately from the number of original articles.

Concerning the publication date, we prioritized the print date for journals that had both print and electronic versions; for example, we used the following text to search for research articles published on '*AIDS*' during 1998-2006 and whose first author was in Japan: *AIDS [Jour] AND journal article [pt] AND Japan [ad] AND 1998:2006 [PPDAT]*.

Additionally, we compared the number of research articles registered in the selected journals to those in the SCI Infectious Disease Category during the period, to ascertain whether the newly selected journals were appropriate to survey a wide range of articles in the field of infectious disease research. We surveyed articles registered in the SCI category in a manner similar to those in the above selected journals.

### Step-5 Analysis of Asian publications

In order to evaluate socioeconomic factors associated with Asian publications, we weighted the number of research articles registered in the newly selected journals during 1998-2006 according to the population and the gross domestic product (GDP) of each country. We obtained annual data for the population and GDP from the online database of the United Nations [36] for Asian countries. Regarding Taiwan, we used the data from the Taiwan Statistical Data Book 2008 published by the Taiwan's Council for Economic Planning and Development [37]. Using the non-parametric correlation statistical test (Spearman's Rank Correlation test), the numbers of research articles were analyzed in relation to the population and GDP. Statistical analyses were performed using PASW Statistics (version 17.0; SPSS Japan Inc., Tokyo, Japan).

## Results

### Journal selection

To screen infectious disease journals (*Step *2 of Figure 1), 264 candidates were selected (see the actual journals in Additional file 1), of which 240 were selected by English key words and 24 by non-English words. The 264 journals were published in 30 countries and written in 12 languages: English, Japanese, Chinese, Korean, French, German, Italian, Spanish, Turkish, Polish, Russian, and Croatian (see 'Language used' in Additional file 1). Subsequently, we selected 100 of the 264 journals, based on the usability of the PubMed database (*Step 3 *of Figure 1, see Table 1 and gray labeled journals in Additional file 1). The 100 journals were published in 18 countries and written in English or 7 non-English languages: Japanese, Chinese, French, German, Spanish, Turkish, and Russian (Additional file 2). Forty-eight of the 100 journals matched the journals in the SCI Infectious Disease Category, except for 8 journals (the journals were outside our analysis condition, see details in the legend to Additional file 2). The remaining 52 journals were originally selected in this study and included 15 Asian journals, which consisted of 3 journals in Japanese and 2 in Chinese, 7 in English, and 3 in both English and Japanese or Chinese. The breakdown of the 100 journals corresponding to categories A to F in Figure 1 was: 21 in A, 35 in B, 16 in C, 17 in D, 2 in E, and 3 in F (94 journals). Six of the 100 journals belonged to 2 categories: 2 in A and B, 2 in B and C, 1 in B and D, and 1 in C and D.

**Table 1:** List of 100 infectious disease journals selected in the study

AIDS	Diagnostic Microbiology and Infectious Disease	Journal of Acquired Immune Deficiency Syndromes (1999)	Malaria Journal
AIDS Patient Care and STDs	Emerging Infectious Diseases	The Japanese Journal of Antibiotics	Médecine et Maladies Infectieuses
The AIDS Reader	Enfermedades Infecciosas y Microbiología Clínica	Japanese Journal of Infectious Diseases	Medical Microbiology and Immunology
AIDS Research and Human Retroviruses	Epidemiology and Infection	Nihon Hansenbyô Gakkai zasshi (Japanese Journal of Leprosy)	Medical Mycology
AIDS Reviews	European Journal of Clinical Microbiology & Infectious Diseases	Nihon Ishinkin Gakkai zasshi (Japanese Journal of Medical Mycology)	Microbes and Infection
American Journal of Infection Control	Expert Review of Vaccines	The Journal of Antibiotics	Microbial Drug Resistance
The American Journal of Tropical Medicine and Hygiene	FEMS Immunology and Medical Microbiology	The Journal of Antimicrobial Chemotherapy	Mycoses
Annals of Tropical Medicine and Parasitology	Genetic Vaccines and Therapy	Journal of Clinical Microbiology	The Pediatric Infectious Disease Journal
Antimicrobial Agents and Chemotherapy	HIV Clinical Trials	Journal of Clinical Virology	Problemy tuberkuleza i boleznei legkikh
Annals of clinical microbiology and antimicrobials	HIV Medicine	Journal of Communicable Diseases	Reviews in Medical Virology
Antiviral Chemistry & Chemotherapy	Human Vaccines	The Journal of Hospital Infection	Scandinavian Journal of Infectious Diseases
Antiviral Research	Indian Journal of Leprosy	Journal of Immune Based Therapies and Vaccines	Sexually Transmitted Diseases
Antiviral Therapy	Indian Journal of Medical Microbiology	The Journal of Infection	Sexually Transmitted Infections
Biologicals	Infection	Journal of Infection and Chemotherapy	Surgical Infections
BMC Infectious Diseases	Infection and Immunity	The Journal of Infectious Diseases	The Brazilian Journal of Infectious Diseases
Canada communicable disease report	Infection Control and Hospital Epidemiology	Journal of Medical Microbiology	Transplant Infectious Disease
Clinical and Vaccine Immunology	Infectious Disease Clinics of North America	Journal of Medical Virology	Travel Medicine and Infectious Disease
Clinical Infectious Diseases	Infectious Diseases in Obstetrics and Gynecology	Journal of Microbiological Methods	Tropical Medicine & International Health
Clinical Microbiology and Infection	Infectious Disorders Drug Targets	Wei mian yu gan ran za zhi	Tuberculosis
Clinical Microbiology reviews	International Journal of Antimicrobial Agents	(Journal of Microbiology, Immunology, and Infection)	Tuberkuloz ve toraks
Communicable diseases intelligence	International Journal of Hygiene and Environmental Health	Journal of Vector-borne Diseases	Vaccine
Comparative Immunology, Microbiology and Infectious Diseases	International Journal of Infectious Diseases	Journal of Viral Hepatitis	Vector-borne and Zoonotic Diseases
Current HIV Research	International Journal of Medical Microbiology	Journal of Virological Methods	Zhonghua jie he he hu xi za zhi (Chinese Journal of Tuberculosis and Respiratory Diseases)
Current Infectious Disease reports	International Journal of STD & AIDS	Kansenshogaku zasshi (The Journal of the Japanese Association for Infectious Diseases)	Zhonghua shi yan he lin chuang bing du xue za zhi (Chinese Journal of Experimental and Clinical Virology)
Current Opinion in Infectious Diseases	The International Journal of Tuberculosis and Lung Disease	Kekkaku (Tuberculosis)	
		The Lancet Infectious Diseases	
		Leprosy Review	

### Usability analysis of the newly selected journals

**Figure 2:**
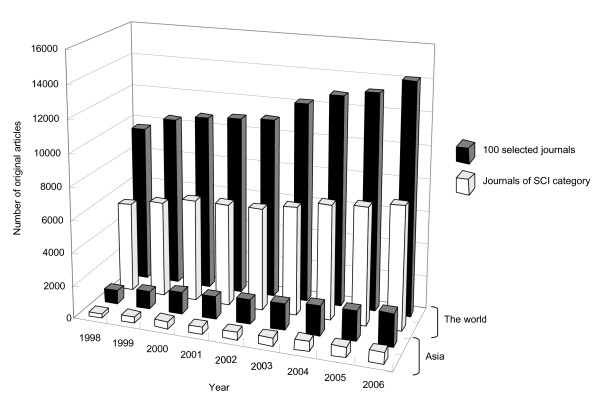
**Number of original articles originating from Asia and worldwide**.

**Figure 3:**
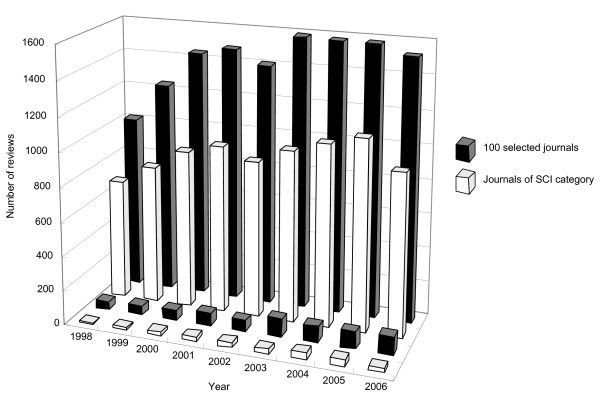
**Number of reviews originating from Asia and worldwide**.

In order to ascertain whether the 100 newly selected journals could survey a wide range of infectious disease research articles, we compared the 100 journals and the journals of the SCI Infectious Disease Category from the viewpoint of the difference in the actual number of Asian and worldwide research articles, and the proportion of Asian research articles relative to the world total. Regarding the actual number of research articles, a survey of the 100 selected journals showed more articles than the SCI Infectious Disease Category. The 100 journals published 14,156 research articles (original articles and reviews) of Asian origin and 118,158 of the world total, whereas the journals in the SCI category published 4,621 of Asian origin and 66,518 of the world total during 1998-2006. Regarding the total number of original articles, the 100 journals published about 3-fold of the Asian articles (the actual numbers registered in the 100 journals and the SCI category were 13,452 and 4,412, hereinafter, as described in this paragraph) and 1.8-fold of the worldwide articles (105,308 and 58,424) (Additional file 3 and 4). For the number of reviews, the 100 journals published about 3.4-fold of the Asian articles (705 and 209) and 1.5-fold of the worldwide articles (12,850 and 8,094). Based on the number of original articles published each year from 1998 to 2006, a survey of the 100 journals showed about 2.9- to 3.3-fold (Asian countries) and 1.7- to 1.9-fold (world) higher than in the SCI category (Figure 2 and 3, Additional file 3 and 4). Regarding reviews, the survey of the 100 journals was about 2.4- to 6.9-fold (Asian countries) and 1.5- to 1.7-fold (world) higher than in the SCI category.

Concerning the proportion of Asian articles relative to the world total, it was revealed that a survey of the 100 journals showed a consistently higher percentage than the SCI Infectious Disease Category during 1998-2006. The total of Asian research articles accounted for 12% of the world total in the survey of 100 journals (actual numbers of Asian and worldwide research articles were 14,156 and 118,158, hereinafter, as described in this paragraph) (Additional file 3) and 6.9% in the survey of SCI Infectious Disease Category (4,621 and 66,518) (Additional file 4). Each year during the study period, the proportion of original articles of Asian origin relative to the world total was about 8.6-14.2% in the 100 journals and 4.7-9.3% in the SCI category, and that of reviews of Asian origin was about 4.2-6.9% in the 100 journals and 1.0-3.9% in the SCI category (Additional file 3 and 4).

### Asian publications in originally selected journals

Subsequently, we surveyed the number of research articles of Asian origin on a country-by-country basis, using the 100 selected journals. It was revealed that Japan had the highest percentage of articles in Asian countries, followed by China, India and Taiwan in descending order during 1998-2006 (Table 2). Additionally, Japan, India and Taiwan produced relatively large numbers of reviews in comparison with other countries (see the column 'Relative to the total number of reviews (RV) of 11 Asian countries' in Table 2). On the other hand, Singapore, Thailand and the Philippines had a high ratio of reviews in their domestic total number of research articles regardless of the relatively low ratio of reviews by Asian countries (see the column 'Relative to the domestic total number' in Table 2).

**Table 2:** Relative comparison of the number of Asian research articles during 1998--2006

	Relative to the domestic total number		Relative to the total number of OR, RV or OR+RV of 11 Asian countries			Relative to the total number of OR, RV or OR+RV of the world		
	**OR/OR+ RV**	**RV/OR+ RV**	**OR/OR**	**RV/RV**	**OR+RV/OR+RV**	**OR/OR**	**RV/RV**	**OR+RV/OR+RV**

Japan	93.3	6.7	46.1	63.0	46.9	5.9	3.5	5.6
China	98.5	1.5	16.3	4.7	15.7	2.1	0.3	1.9
India	95.8	4.2	16.3	13.5	16.1	2.1	0.7	1.9
Taiwan	95.3	4.7	9.9	9.4	9.9	1.3	0.5	1.2
Korea	97.8	2.3	5.9	2.7	5.8	0.8	0.1	0.7
Singapore	91.0	9.0	1.9	3.5	2.0	0.2	0.2	0.2
Malaysia	94.9	5.1	1.0	1.0	1.0	0.1	0.1	0.1
Indonesia	98.3	1.7	0.9	0.3	0.8	0.1	< 0.1	0.1
Vietnam	96.4	3.6	0.8	0.6	0.8	0.1	< 0.1	0.1
Thailand	92.3	7.7	0.5	0.9	0.6	0.1	< 0.1	0.1
Philippines	93.8	6.2	0.5	0.6	0.5	0.1	< 0.1	0.1

Asian countries (above 11 countries)	NA	NA	NA	NA	NA	12.8	5.5	12.0

The numeric data show %.

OR, original articles; RV, reviews; NA, not applicable.

**Figure 4:**
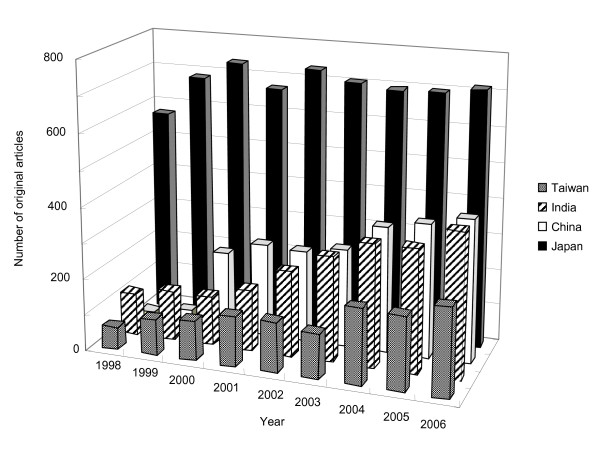
**Number of original articles originating from 4 Asian countries**.

Looking at the change in the number of research articles from 1998-2006, China, Taiwan, and India showed high increase rates of original articles; the numbers in 2006 were more than about 18-fold (China), 3.9-fold (Taiwan), and 3.5-fold (India) than in 1998 (Figure 4, see the data corresponding to each country in Additional file 3). The number of original articles produced in Japan was stable (the number in 2006 was about 1.2-fold that in 1998), regardless of having the greatest total number of articles by Asian countries. For Korea, original articles increased markedly (under 30 articles in 1998 to over 150 in 2006), and articles produced by other countries, Singapore, Malaysia, Indonesia, Vietnam, Thailand, and the Philippines, increased slightly or were stable. Concerning the number of reviews produced in 11 Asian countries, no noticeable increase was revealed during the study period.

**Figure 5:**
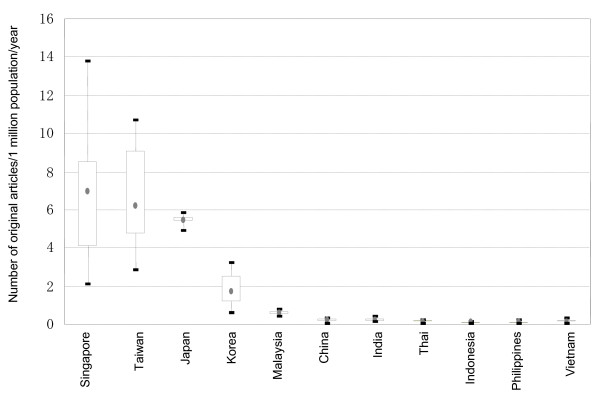
**Asian publication of original articles by population**. Upper horizontal line, dot, and lower horizontal line in the box represent the first, second (median), and third quartiles. Whiskers represent extension of values up and down.

As a further analysis of Asian publications, the number of research articles was compared to the population and GDP. For the population, the ratio of the number of original articles to the population of individual Asian countries showed a median value of 0.2 publications/1 million population/year (range, 0.1-6.9) during 1998-2006. Using population-adjusted ratios, Singapore (median value of 6.9) and Taiwan (6.2) were most productive (Figure 5). The ratio of the number of reviews showed a median value of < 0.1 publications/1 million population/year (range, 0-0.5). When adjusting the production of reviews according to population, Singapore ranked first (0.5), followed by Japan (0.4) and Taiwan (0.3). There was no statistically significant correlation between the average population and the number of original articles (Spearman's correlation coefficient = 0.255, *p *= 0.450) or reviews (Spearman's correlation coefficient = 0.055, *p *= 0.873) in 11 Asian countries during 1998-2006.

**Figure 6:**
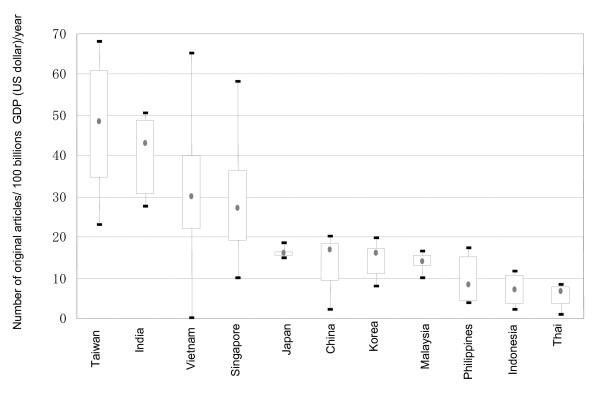
**Asian publication of original articles by GDP**. Upper horizontal line, dot, and lower horizontal line in the box represent the first, second (median), and third quartiles. Whiskers represent extension of values up and down.

Regarding the ratio of the number of original articles to the GDP, the median value was 16.6 publications/100 billion GDP/year (range, 6.4-48.3). For GDP-adjusted ratios, Taiwan (48.3) and India (42.9) were highly productive (Figure 6). The ratio of the number of reviews showed a median value of 0.6 publications/1 million population/year (range, 0-3.3). When adjusting production of reviews according to the GDP, Vietnam (3.3), Singapore (2.5) and Taiwan (2.4) were most productive. There was a statistical correlation between the average GDP and the number of original articles (Spearman's correlation coefficient = 0.864, *p *= 0.001) or reviews (Spearman's correlation coefficient = 0.691, *p *= 0.019) in Asian countries.

## Discussion

Our study has demonstrated that a survey method using 100 selected journals is beneficial to grasp the overall trends in infectious disease research in comparison with previous bibliometric studies based on journals registered in the SCI Infectious Disease Category. This is derived from the findings that a survey of 100 journals showed not only more research articles originating from both Asian countries and worldwide, but also a higher proportion of Asian articles relative to the world total than in the SCI Infectious Disease Category. The survey succeeded in analyzing Asian trends in infectious disease research by identifying research articles published even in regional and non-English journals.

To our knowledge, the trend per Asian country was analyzed for the first time in this study. Japan was considered to be the leading country in the field of infectious disease research in Asia because it had the highest percentage of both original articles and reviews during 1998-2006 in the survey of 100 journals. Additionally, China, India, and Taiwan were assumed to have markedly elevated productivity in research, and research results from the latter 2 countries were better appreciated among Asian countries from the viewpoint of the higher rate of original articles and reviews than other Asian countries (except for Japan). On the other hand, Singapore and Taiwan ranked high when the population and GDP were taken into account. Regarding Singapore, the analysis result is comparable to the previous report that the country ranked higher than Japan and Taiwan in the field of biomedical research when adjusting the number of publications to the population [38]. Further analysis with science and technology indicators as the number of researchers and the public expenditure on research and development, as shown in previous reports [38,39], is considered to be favorable for our study, however, such analysis was not conducted. This is because annual data from the 11 Asian countries were not fully available during the study period, even using the databases of international organizations such as the Main Science and Technology Indicators of the OECD [40] and the Science and Technology Indicators of the ASEAN STI/TCI [41].

Regardless of improved journal selection, as described above, our method has several methodological limitations. First, the keyword setting for journal selection has limitations; the 100 newly selected journals are not equivalent to existing full infectious disease journals. This is attributed to the fact that we set representative keywords for journal selection in several languages used by many people throughout the world, but did not cover all the keywords related to infectious diseases research. Additionally, our procedure for journal selection was not entirely automated, requiring not only keywords, but also professional judgment (see Figure 1F). Given these observations, our method may be difficult to generally apply to a survey of infectious disease research around the world; however, we think that our journal selection has demonstrated the prospect for a more exhaustive survey of infectious disease research.

Concerning the keyword setting for journal selection, our method has another limitation. Since we selected 100 journals on the basis of keywords directly linked to infectious disease control, we missed articles published in general scientific and medical journals, such as 'Nature', published by the Nature Publishing Group, and 'Science', published by the American Association for the Advancement of Science (AAAS), and general medical journals, such as the 'New England Journal of Medicine', published by Massachusetts Medical Society. This limitation of journal selection might be common because this disadvantage was also noted in the previous study [22]. However, we believe that the number of missed articles did not significantly affect our study results because our survey scheme was designed to understand the overall trends in infectious disease research by obtaining the relative numbers of research articles per country or region, not to strictly count the absolute numbers of articles.

An additional limitation relates to the survey of author affiliation in the PubMed database. Since we based the affiliation on country name, we missed research articles whose author's affiliations were recorded as the names of the city, district, university or institution (not country). The reason comes from the fact that such articles were reported previously [12,26,42] and additional articles could be retrieved by applying names of cities and institutions in a country [12,42]. However, we think that the preference for country name as the author affiliation is appropriate as far as this study goes because research articles originating from each Asian country should be counted under fixed condition for an international comparison of research productivity. Moreover, it is difficult to establish sufficient names for a city or institution; taking Japan as an example, there are numerous research institutions, universities and other organizations engaged in research on infectious diseases across the country. That said, as further issues, we should develop a setting for author affiliation corresponding to various countries and regions.

As further issues, our study should introduce qualitative analysis of infectious disease research articles, based on two viewpoints. One intends to survey the content characteristics of the disease research, such as targeted diseases, methodology used (epidemiological, pathological, etc.) and so on. The analysis will contribute to grasping the outline of the research outcome and formulate a further strategy for research. The other is designed to analyze the scientific quality of the research. As an effective and valuable method for analyzing scientific quality, a measurement of the IF has been applied globally; nevertheless, the measurement is considered to have an English language bias [43]. On this basis, the current method of IF measurement might be poorly adapted for qualitative analysis of regional and non-English journals; therefore, a new method for expanding the versatility of qualitative analysis should be developed hereafter.

## Conclusion

We present a new journal selection to survey articles of infectious disease research. The 100 selected journals contribute to quantitative survey of research articles in not only international, but also regional and non-English journals, with little bias among countries and regions. We suggest that surveying these 100 journals is more beneficial than the SCI Infectious Disease Category, because it identifies more research articles and avoids underestimation of the numbers of articles in regional and non-English journals. Our survey method may require further development; nevertheless, the method provides an effective tool for grasping overall trends in infectious disease research around the world.

## Competing interests

The authors declare that they have no competing interests.

## Authors' contributions

HTO conceived of the study, formulated its design, carried out the data collection and drafted the manuscript. KO participated in the design of the study and statistically analyzed the data. NO participated in the design of the study and coordination. All authors read and approved the final manuscript.

## Pre-publication history

The pre-publication history for this paper can be accessed here:

http://www.biomedcentral.com/1471-2288/9/67/prepub

## Supplementary Material

Additional file 1 **Infectious disease journals selected initially**. The journal list shows 264 candidates for infectious disease journals. Click here for file

Additional file 2 **Details about the 100 newly selected journals**. The data shows details about the infectious disease journals selected in this study. Click here for file

Additional file 3 **Number of infectious disease research articles in the 100 journals**. The data shows the number of infectious disease research articles published in the 100 newly selected journals. Click here for file

Additional file 4 **Number of infectious disease research articles in the SCI Infectious Disease Category**. The data shows the number of infectious disease research articles published in the journals registered in the 'Infectious Disease Category' of the Science Citation Index Expanded™. Click here for file
