# Supplementary material for: New journal selection for quantitative survey of infectious disease research: application for Asian trend analysis
Source: BMC Med Res Methodol. 2009 Oct 6;9:67. doi: 10.1186/1471-2288-9-67 (PMC2766390; doi:10.1186/1471-2288-9-67)
Supplement: Additional file 1 — Infectious disease journals selected initially. The journal list shows 264 candidates for infectious disease journals. [file 1471-2288-9-67-S1.PDF]

Additional file 1 Infectious disease journals selected initially

Language of key word for journal selection E; English NE; non-English	Journal title	Language used*	Publishing country	Reason for not being listed as the infectious disease journals selected finally (except for gray labeled journals)
E	Advances in pediatric infectious diseases	English	US	Inactive in January 2008
E	African journal of AIDS research	English	Tanzania	Not registered in the the PubMed
E	Aging: Immunology and infectious disease	English	US	Inactive in January 2008
E	AIDS†	English	UK	
E	AIDS action	English	UK	Inactive in January 2008
E	AIDS alert	English	US	Not registered in the SCI category ‡
E	AIDS analysis africa	English	South Africa	Inactive in January 2008
E	AIDS and behavior	English	US	Not registered in the SCI category ‡
E	AIDS and public policy journal	English	US	Not registered in the SCI category ‡
E	AIDS care - Psychological and socio-medical aspects of AIDS/HIV	English	UK	Not registered in the SCI category ‡
E	AIDS clinical care	English	US	Not registered in the SCI category ‡
E	AIDS clinical review	English	US	Inactive in January 2008
E	AIDS education and prevention	English	US	Not registered in the SCI category ‡
E	AIDS health promotion exchange / World Health Organization, Global Programme on AIDS, Health Promotion Unit	English	Switzerland	Inactive in January 2008
E	AIDS patient care	English	US	Inactive in January 2008

E	AIDS patient care and STDs	English	US	
E	AIDS policy & law	English	US	Not registered in the SCI category ‡
E	AIDS reader	English	US	
E	AIDS research	English	US	Inactive in January 2008
E	AIDS research and human retroviruses	English	US	
E	AIDS research and therapy	English	UK	Not registered in the SCI category ‡
E	AIDS reviews	English	Spain	
E	AIDS treatment news	English	US	Not registered in the SCI category ‡
E	AIDS weekly	English	US	Not registered in the SCI category ‡
E	AIDS weekly plus	English	US	Not registered in the SCI category ‡
E	AIDS, Supplement	English	UK	Not registered in the SCI category ‡
E	AIDS/STD health promotion exchange	English	Netherlands	Not registered in the SCI category ‡
E	Aidscaptions	English	US	Inactive in January 2008
E	AIDS-Forschung : AIFO = Acquired immune deficiency syndrome research	German	Germany	Not registered in the SCI category ‡
E	AIDSlink : Eastern, Central & Southern Africa	English	Tanzania	Not registered in the SCI category ‡
E	American journal of infection control	English	US	
E	American journal of tropical medicine and hygiene	English	US	
E	Annals of clinical microbiology and antimicrobials	English	UK	
E	Annals of tropical medicine and parasitology	English	UK	
E	Antibiotics and chemotherapy	English	Switzerland	Inactive in January 2008

E	Antibiotiki i meditsinskaia biotekhnologiia = Antibiotics and Medical Biotechnology / Ministerstvo meditsinskoi promyshlennosti SSSR	Russian	Russia	Inactive in January 2008
E	Antimicrobial agents and chemotherapy	English	US	
E	Antimicrobial agents annual	English	Netherlands	Inactive in January 2008
E	Antimicrobics and infectious diseases newsletter	English	Netherlands	Inactive in January 2008
E	Anti-infective agents in medicinal chemistry	English	Netherlands	Not registered in the PubMed
E	Antiinfective drugs and chemotherapy	English	Germany	Inactive in January 2008
E	Antiviral chemistry and chemotherapy	English	UK	
E	Antiviral chemistry and chemotherapy, Supplement	English	UK	Inactive in January 2008
E	Antiviral research	English	Netherlands	
E	Antiviral therapy	English	UK	
E	Archives of AIDS research	English	US	Not registered in the SCI category ‡
E	Archives of STD/HIV research	English	US	Inactive in January 2008
E	Bailliere's clinical infectious diseases	English	UK	Inactive in January 2008
E	BETA bulletin of experimental treatments for AIDS:A publication of the San Francisco AIDS Foundation	English	US	Not registered in the SCI category ‡
E	Biologicals:Journal of the International Association of Biological Standardization UK	English	UK	
E	BMC infectious diseases	English	UK	
E	British journal of infection control	English	UK	Not registered in the PubMed

E	Bulletin - National Tuberculosis and Respiratory Disease Association	English	US	Inactive in January 2008
E	Bulletin of the International Union against Tuberculosis	English, French	France	Inactive in January 2008
E	Bulletin of the International Union Against Tuberculosis and Lung Disease	English	France	Inactive in January 2008
E	Canada communicable disease report = Releve des maladies transmissibles au Canada	English French	Canada	
E	Canadian AIDS news	English, French	Canada	Not registered in the SCI category ‡
E	Canadian HIV/AIDS policy & law review / Canadian HIV/AIDS Legal Network	English, French	Canada	Not registered in the SCI category ‡
E	Canadian HIV-AIDS policy & law newsletter / Canadian HIV-AIDS Legal Network = Reseau juridique canadien VIH-SIDA	English, French	Canada	Not registered in the SCI category ‡
E	Canadian journal of infectious diseases (continues as Canadian journal of infectious diseases and medical microbiology)	English, French	Canada	Inactive in January 2008
E	Canadian journal of infectious diseases and medical microbiology	English	Canada	Not suitable for our study because the journal involves a considerable number of non-research articles, such as clinical vignettes and guidelines, and so on
E	CDC AIDS weekly	English	US	Not registered in the SCI category ‡
E	Chinese journal of antibiotics	Chinese	China	Articles are not available in the PubMed§
E	Chinese journal of infection and chemotherapy	Chinese	China	Not registered in the PubMed

E	Clinical and vaccine immunology	English	US	
E	Clinical infectious diseases	English	US	
E	Clinical microbiology and infection	English	France	
E	Clinical microbiology and infection, Supplement	English	France	Not registered in the PubMed
E	Clinical microbiology newsletter	English	US	Articles are not available in the PubMed§
E	Clinical microbiology reviews	English	US	
E	Communicable disease and public health/PHLS	English	UK	Inactive in January 2008
E	Communicable disease report. CDR review	English	UK	Inactive in January 2008
E	Communicable disease report. CDR weekly	English	UK	Inactive in January 2008
E	Communicable diseases intelligence	English	Australia	
E	Comparative immunology, microbiology and infectious diseases	English, French	UK	
E	CPD infection	English	UK	Not registered in the PubMed
E	Critical path AIDS project	English	US	Not registered in the SCI category ‡
E	Current clinical topics in infectious diseases	English	US	Inactive in January 2008
E	Current drug targets - infectious disorders	English	Netherlands	Inactive in January 2008
E	Current HIV research	English	Netherlands	
E	Current HIV/AIDS reports	English	US	Not registered in the SCI category ‡
E	Current infectious disease reports	English	US	
E	Current opinion in HIV and AIDS	English	US	Not registered in the SCI category ‡

E	Current opinion in infectious diseases	English	US	
E	Current topics in medical mycology	English	Spain	Inactive in January 2008
E	Diagnostic microbiology and infectious disease	English	US	
E	Emerging infectious diseases	English	US	
E	Enfermedades infecciosas y microbiologia clinica	Spanish	Spain	
E	Epidemiology and infection	English	UK	
E	Euro surveillance : Bulletin europeen sur les maladies transmissibles = European communicable disease bulletin	English	Sweden	Bulletins are excluded from our survey
E	European journal of clinical microbiology	English	Germany	Inactive in January 2008
E	European journal of clinical microbiology and infectious diseases	English	Germany	
E	Expert review of vaccines	English	UK	
E	FEMS immunology and medical microbiology	English	UK	
E	Genetic vaccines and therapy	English	UK	
E	GMHC treatment issues : The Gay Men's health crisis newsletter of experimental AIDS therapies	English	US	Not registered in the SCI category ‡
E	Gruzlica i choroby pluc; Tuberculosis et pneumonologia	Polish	Poland	Inactive in January 2008
E	Hindustan antibiotics bulletin	English	India	Inactive in January 2008
E	HIV and AIDS review	English	Poland	Not registered in the SCI category ‡
E	HIV clinical trials	English	US	

E	HIV clinician / Delta Region AIDS Education & Training Center	English	US	Not registered in the SCI category ‡
E	HIV medicine	English	UK	
E	HIV nursing	English	UK	Not registered in the SCI category ‡
E	HIV prevention plus! : The newsletter of the Canadian HIV/AIDS Clearinghouse	English, French	Canada	Not registered in the SCI category ‡
E	HIV/AIDS policy & law review / Canadian HIV/AIDS legal network.	English, French	Canada	Not registered in the SCI category ‡
E	Hospital infection control	English	US	Articles published in 1984 or later are not available in the PubMed
E	Human vaccines	English	US	
E	IAVI report : Newsletter on international AIDS vaccine research.	English	US	Not registered in the SCI category ‡
E	Immunology and infectious diseases	English	UK	Inactive in January 2008
E	Indian journal of leprosy	English	India	
E	Indian journal of medical microbiology	English	India	
E	Indian journal of medical research - Section A infectious diseases	English	India	Inactive in January 2008
E	Indian journal of medical research - Section B biomedical research other than infectious diseases	English	India	Not suitable for our study because the journal targets non-communicable diseases
E	Indian journal of tuberculosis	English	India	Articles are registered in the PubMed in 2007 or later
E	Infection	English German	Germany	

E	Infection and immunity	English	US	
E	Infection control	English	US	Inactive in January 2008
E	Infection control and hospital epidemiology	English	US	
E	Infection control Canada	English, French	Canada	Inactive in January 2008
E	Infection, genetics and evolution	English	Netherlands	Not suitable for our study because the journal focuses on basic research as the genetics of hosts and microorganisms, and so on
E	Infection, Supplement	English German	Germany	Not registered in the PubMed
E	Infections in medicine	English	US	Only one article published in 1993 is available in the PubMed
E	Infections in surgery	English	US	Inactive in January 2008
E	Infectious agents and disease	English	US	Inactive in January 2008
E	Infectious disease clinics of North America	English	US	
E	Infectious diseases in clinical practice	English	US	Not registered in the PubMed
E	Infectious diseases in obstetrics and gynecology	English	Egypt	
E	Infectious disorders - Drug targets	English	United Arb Emirates	
E	International antiviral news	English	UK	Inactive in January 2008
E	International journal of antimicrobial agents	English	Netherlands	
E	International journal of hygiene and environmental health	English	Germany	

E	International journal of infectious diseases	English	Canada	
E	International journal of leprosy	English	US	Inactive in January 2008
E	International journal of leprosy and other mycobacterial diseases	English	US	Inactive in January 2008
E	International journal of medical microbiology	English	Germany	
E	International journal of medical microbiology, Supplement	English	Germany	Not registered in the PubMed
E	International journal of STD and AIDS	English	UK	
E	International journal of tuberculosis and lung disease	English	France	
E	Japanese journal of antibiotics	Japanese	Japan	
E	Japanese journal of infectious diseases	English	Japan	
E	Japanese journal of leprosy	Japanese English	Japan	
E	Japanese journal of medical mycology	Japanese English	Japan	
E	Journal of antibacterial and antifungal agents, Japan	Japanese	Japan	Inactive in January 2008
E	Journal of antibiotics	English	Japan	
E	Journal of antimicrobial chemotherapy	English	UK	
E	Journal of acquired immune deficiency syndromes (1999)	English	US	
E	Journal of clinical microbiology	English	US	
E	Journal of clinical virology	English	Netherlands	
E	Journal of communicable diseases	English	India	

E	Journal of HIV therapy	English	UK	Not registered in the SCI category ‡
E	Journal of HIV/AIDS and social services	English	US	Not registered in the SCI category ‡
E	Journal of HIV/AIDS prevention and education for adolescents and children	English	US	Not registered in the SCI category ‡
E	Journal of HIV/AIDS prevention in children and youth	English	US	Not registered in the SCI category ‡
E	Journal of hospital infection	English	UK	
E	Journal of hygiene epidemiology microbiology and immunology	Mutiple languages	Czech Republic	Inactive in January 2008
E	Journal of immune based therapies and vaccines	English	UK	
E	Journal of infection	English	UK	
E	Journal of infection and chemotherapy	English	Japan	
E	Journal of infectious disease pharmacotherapy	English	US	Not registered in the PubMed
E	Journal of infectious diseases	English	US	
E	Journal of medical and veterinary mycology	English	UK	Inactive in January 2008
E	Journal of medical and veterinary mycology, Supplement	English	UK	Inactive in January 2008
E	Journal of medical microbiology	English	UK	
E	Journal of medical virology	English	US	
E	Journal of microbiological methods	English	Netherlands	

E	Journal of microbiology, immunology and infection	Chinese English	China	
E	Journal of neuro-AIDS	English	US	Not registered in the SCI category ‡
E	Journal of the association of nurses in AIDS care	English	US	Not registered in the SCI category ‡
E	Journal of the International Association of Physicians in AIDS Care	English	US	Not registered in the SCI category ‡ Inactive in January 2008
E	Journal of the International Association of Physicians in AIDS Care:JIAPAC [Journal of the International Association of Physicians in AIDS Care (Chicago, Ill. : 2002]	English	US	Not registered in the SCI category ‡
E	Journal of the physicians association for AIDS care	English	US	Not registered in the SCI category ‡
E	Journal of trauma - Injury, infection and critical care	English	US	Not suitable for our study because the journal focuses on trauma
E	Journal of vector borne diseases	English	India	
E	Journal of veterinary medicine series B: Infectious diseases and veterinary public health	English	Germany	Inactive in January 2008
E	Journal of viral hepatitis	English	UK	
E	Journal of virological Methods	English	Netherlands	
E	Kansenshogaku zasshi. The journal of the Japanese Association for Infectious Diseases	Japanese	Japan	
E	Kekkaku	Japanese	Japan	
E	Korean journal of medical mycology	Korean	Korea	Not registered in the PubMed
E	Lancet infectious diseases	English	US	

E	Leprosy in India	English	India	Inactive in January 2008
E	Leprosy review	English	UK	
E	Malaria journal	English	UK	
E	Medecine et maladies infectieuses	French	France	
E	Medical microbiology and immunology	English	Germany	
E	Medical microbiology letters	English	Switzerland	Inactive in January 2008
E	Medical mycology	English	UK	
E	Mediterranean journal of infectious and parasitic diseases	English	UK	Inactive in January 2008
E	Microbes and infection	English	France	
E	Microbial drug resistance	English	US	
E	Mycoses	English	Germany	
E	Newsline (people with AIDS coalition of New York)	English	US	Not registered in the SCI category ‡
E	NIAID AIDS agenda / National Institute of Allergy and Infectious Diseases	English	US	Not registered in the SCI category ‡
E	Nutrition and metabolic disorders in HIV infection	English, Spanish	Spain	Not registered in the SCI category ‡
E	Pacific AIDS alert bulletin / South Pacific Commission	English	New Caledonia	Not registered in the SCI category ‡
E	Pediatric AIDS and HIV infection	English	US	Not registered in the SCI category ‡
E	Pediatric infectious disease	English	US	Inactive in January 2008
E	Pediatric infectious disease journal	English	US	

E	Perspectives in medical virology	English	Netherlands	Articles are not available in the PubMed§
E	Problems of infectious and parasitic diseases	English	Bulgaria	Not registered in the PubMed
E	Problemy HIV i AIDS	Polish	Poland	Not registered in the SCI category ‡
E	Report on pediatric infectious diseases	English	US	Inactive in January 2008
E	Repura. Leprosy	Japanese	Japan	Inactive in January 2008
E	Reviews in medical microbiology	English	UK	Not registered in the PubMed
E	Reviews in medical virology	English	UK	
E	Reviews of infectious diseases	English	US	Inactive in January 2008
E	Sabouraudia journal of medical and veterinary mycology	English	UK	Inactive in January 2008
E	SAfAIDS news : Southern Africa AIDS Information Dissemination Service bulletin	English	Zimbabwe	Not registered in the SCI category ‡
E	Scandinavian journal of infectious diseases	English	Sweden	
E	Scandinavian journal of infectious diseases, Supplement	English	Sweden	Inactive in January 2008
E	Seminars in pediatric infectious diseases	English	US	Inactive in January 2008
E	Seminars in respiratory infections	English	US	Inactive in January 2008
E	Serodiagnosis and immunotherapy in infectious disease	English	US	Inactive in January 2008
E	Sexually transmitted diseases	English	US	
E	Sexually transmitted infections	English	UK	
E	Southern African journal of HIV medicine	English	South Africa	Not registered in the SCI category ‡

E	Surgical infections	English	US	
E	The Brazilian journal of infectious diseases : An official publication of the Brazilian Society of Infectious Diseases	English	Brazil	
E	The Canadian journal of infection control : The official journal of the Community & Hospital Infection Control Association-Canada = Revue canadienne de prevention des infections / Association pour la prevention des infections a l'hopit	English, French	Canada	Inactive in January 2008
E	The Hopkins HIV report : A bimonthly newsletter for healthcare providers / Johns Hopkins University AIDS Service	English	US	Not registered in the SCI category ‡
E	The Journal of antibiotics. Ser. B	Japanese	Japan	Inactive in January 2008
E	Topics in HIV medicine : A publication of the International AIDS Society, USA	English	US	Not registered in the SCI category ‡
E	Transplant infectious disease	English	Denmark	
E	Travel medicine and infectious Disease	English	Netherlands	
E	Tropical medicine & international health	English	UK	
E	Tuberculosis	English	UK	
E	Tubercle	English	UK	Inactive in January 2008
E	Tubercle and lung disease	English	UK	Inactive in January 2008
E	Tuberculosis and respiratory diseases	English	Korea	Not registered in the PubMed
E	Update/National Minority AIDS Council	English	US	Not registered in the SCI category ‡

E	Vaccine	English	Netherlands	
E	Vaccine research	English	US	Inactive in January 2008
E	Vaccines: Children and practice	English	UK	Not registered in the PubMed
E	Vector-borne and zoonotic diseases	English	US	
E	Viral hepatitis reviews	English	UK	Inactive in January 2008
E	Zentralblatt fur Bakteriologie : International journal of medical microbiology	English	Germany	Inactive in January 2008
E	Zentralblatt fur Bakteriologie, Mikrobiologie, und Hygiene. Series A, Medical microbiology, infectious diseases, virology, parasitology	English, German	Germany	Inactive in January 2008
E	Zhonghua jie he he hu xi za zhi=Zhonghua jiehe he huxi zazhi=Chinese journal of tuberculosis and respiratory diseases	Chinese	China	
E	Zhonghua shi yan he lin chuang bing du xue za zhi=Zhonghua shiyan he linchuang bingduxue zazhi=Chinese journal of experimental and clnical virology	Chinese	China	
E	Zoonoses and public health	English	Germany	The journal has been published since 2007
NE	Acta tuberculosea et pneumologica Belgica	Multiple languages, English	Belgium	Inactive in January 2008
NE	Acta tuberculosea et pneumologica Scandinavica	English	Denmark	Inactive in January 2008
NE	Antibiotiques	French	France	Not registered in the PubMed
NE	Beitrage zur Klinik und Erforschung der Tuberkulose und der Lungenkrankheiten	German	Germany	Inactive in January 2008

NE	Enfermedades infecciosas y microbiologia	Spanish, English	Spain	Inactive in January 2008
NE	Enfermedades infecciosas y microbiologia clinica monografias	Spanish	Spain	Not registered in the PubMed
NE	Giornale italiano della tubercolosi e delle malattie del torace	Italian	Italy	Inactive in January 2008
NE	Immunitat und Infektion	German	Germany	Inactive in January 2008
NE	Krankenhaushygiene und Infektionsverhutung	German	Germany	Not registered in the PubMed
NE	Lotta contro la tubercolosi	Italian	Italy	Inactive in January 2008
NE	Praxis der Pneumologie vereinigt mit der Tuberkulosearzt	German	Germany	Inactive in January 2008
NE	Problemy tuberkuleza	Russian	Russia	Inactive in January 2008
NE	Problemy tuberkuleza i bolezneі legkikh	Russian	Russia	
NE	Revue de tuberculose et de pneumologie	French	France	Inactive in January 2008
NE	Tuberkuloz ve toraks	Turkish	Turkey	
NE	Tuberkuloza	Croatian	Serbia	Inactive in January 2008
NE	ZAC Zeitschrift für Antimikrobielle Antineoplastische Chemotherapie	German	Germany	Inactive in January 2008
NE	Zeitschrift für Tuberkulose und Erkrankungen der Thoraxorgane	German	Germany	Inactive in January 2008
NE	Zentralblatt für Bakteriologie Parasitenkunde Infektionskrankheiten und Hygiene Zweite Abteilung	German	Germany	Inactive in January 2008

NE	Zentralblatt für Bakteriologie, Parasitenkunde, Infektionskrankheiten und Hygiene. 1. Abt. Medizinisch-hygienische Bakteriologie, Virusforschung und Parasitologie. Originale	German	Germany	Inactive in January 2008
NE	Zentralblatt für Bakteriologie, Parasitenkunde, Infektionskrankheiten und Hygiene. Erste Abteilung Originale. Reihe A: Medizinische Mikrobiologie und Parasitologie	German, English	Germany	Inactive in January 2008
NE	Zentralblatt für Bakteriologie, Parasitenkunde, Infektionskrankheiten und Hygiene. Erste Abteilung Originale. Reihe B: Hygiene, Betriebshygiene, präventive Medizin	German	Germany	Inactive in January 2008
NE	Zentralblatt für Bakteriologie, Parasitenkunde, Infektionskrankheiten und Hygiene. Zweite naturwissenschaftliche Abt.: Allgemeine, landwirtschaftliche und technische Mikrobiologie	German, English	Germany	Inactive in January 2008
NE	Zentralblatt für Bakteriologie. 1. Abt. Originale. A: Medizinische Mikrobiologie, Infektionskrankheiten und Parasitologie	German, English	Germany	Inactive in January 2008

*Information of the languages were obtained from the PubMed database and the National Library of Medicine (NLM) Catalog of the National Center for Biotechnology Information.
†Gray labeled journals are the selected 100 infectious disease journals.
‡Journals regarding HIV/AIDS were selected according to the 'Infectious Disease Category' of the Science Citation Index Expanded^{TM} (the SCI category)

§Journal is registered, but articles in the journal are not available in the PubMed
