# Supplementary material for: New journal selection for quantitative survey of infectious disease research: application for Asian trend analysis
Source: BMC Med Res Methodol. 2009 Oct 6;9:67. doi: 10.1186/1471-2288-9-67 (PMC2766390; doi:10.1186/1471-2288-9-67)
Supplement: Additional file 2 — Details about the 100 newly selected journals. The data shows details about the infectious disease journals selected in this study. [file 1471-2288-9-67-S2.PDF]

Additional file 2 Details about the 100 newly selected journals

Journal title*	Registered in Scopus database[†] [33]	Registered in the 'Infectious Disease Category' of the Science Citation Index Expanded^{TM} [32][‡]	Criteria for journal selection (upper)[§], Study period (middle) and publication media (lower)	Publishing country, language[¶], etc.
AIDS (London, England)[#]	○	○**	A<br>1998–2006<br>Print ver.[††]	UK<br>English
AIDS Patient Care and STDs	○	○	A&B<br>1998–2006<br>Print ver.	US<br>English
The AIDS Reader	○	○	A<br>1998–2006<br>Print ver.	US<br>English
AIDS Research and Human Retroviruses	○	○	A<br>1998–2006<br>Print ver.	US<br>English
AIDS Reviews	○	○	A<br>1998–2006<br>Print ver.	Spain<br>English
American Journal of Infection Control	○	○	B<br>1998–2006<br>Print ver.	US<br>English

The American Journal of Tropical Medicine and Hygiene	○		F<br>1998–2006<br>Print ver.	US<br>English
Annals of Tropical Medicine and Parasitology	○		C<br>1998–2006<br>Print ver.	UK<br>English
Antimicrobial Agents and Chemotherapy	○		D<br>1998–2006<br>Print ver.	US<br>English
Annals of clinical microbiology and antimicrobials	○		C&D<br>2002–2006<br>Electronic ver.[‡‡]	UK<br>English
Antiviral Chemistry & Chemotherapy	○		D<br>1998–2006<br>Print ver.	UK<br>English
Antiviral Research	○		D<br>1998–2006<br>Print ver.	Netherlands<br>English
Antiviral Therapy	○	○	D<br>1998–2006<br>Print ver.	UK<br>English
Biologicals: Journal of the International Association of Biological Standardization	○		D<br>1998–2006<br>Print ver.	UK<br>English

UK				
BMC Infectious Diseases	○	○	B<br>2001–2006<br>Electronic ver.	UK<br>English
Canada communicable disease report = Releve des maladies transmissibles au Canada	○		B<br>1998–2006	Canada<br>English<br>French
Clinical and Vaccine Immunology : CVI	○	○	D<br>2006<br>Print ver.	US<br>English
Clinical Infectious Diseases : An official publication of the Infectious Diseases Society of America	○	○	B<br>1998–2006<br>Print ver.	US<br>English
Clinical Microbiology and Infection: The official publication of the European Society of Clinical Microbiology and Infectious Diseases	○	○	C<br>1998–2006<br>Print ver.	France<br>English
Clinical Microbiology reviews	○		C<br>1998–2006<br>Print ver.	US<br>English

Communicable diseases intelligence	○		B<br>1998–2006<br>Print ver.	Australia<br>English
Comparative Immunology, Microbiology and Infectious Diseases	○		B<br>1998–2006<br>Print ver.	UK<br>English, French
Current HIV Research	○	○	A<br>2003–2006<br>Print ver.	Netherlands<br>English
Current Infectious Disease reports	○		B<br>1999–2006<br>Print ver.	US<br>English
Current Opinion in Infectious Diseases	○	○	B<br>1998–2006<br>Print ver.	US<br>English
Diagnostic Microbiology and Infectious Disease	○	○	B&C<br>1998–2006<br>Print ver.	US<br>English
Emerging Infectious Diseases	○	○	B<br>1998–2006<br>Print ver.	US<br>English
Enfermedades Infecciosas y Microbiología Clínica	○	○	C<br>1998–2006<br>Print ver.	Spain<br>Spanish

Epidemiology and Infection	○	○	B<br>1998–2006<br>Print ver.	UK<br>English
European Journal of Clinical Microbiology & Infectious Diseases: Official publication of the European Society of Clinical Microbiology	○	○	B&C<br>1998–2006<br>Print ver.	Germany<br>English
Expert Review of Vaccines	○		D<br>2002–2006<br>Print ver.	UK<br>English
FEMS Immunology and Medical Microbiology	○	○	C<br>1998–2006<br>Print ver.	UK<br>English
Genetic Vaccines and Therapy	○		D<br>2003–2006<br>Electronic ver.	UK<br>English
HIV Clinical Trials	○	○	A<br>2000–2006<br>Print ver.	US<br>English
HIV Medicine	○	○	A<br>1999–2006<br>Print ver.	UK<br>English
Human Vaccines	○		D	US

			2005–2006<br>Print ver.	English
Indian Journal of Leprosy	○		A<br>1998–2006<br>Print ver.	India<br>English<br>Asian journal
Indian Journal of Medical Microbiology	○		C<br>1998–2006<br>Print ver.	India<br>English<br>Asian journal
Infection	○	○	B<br>1998–2006<br>Print ver.	Germany<br>English<br>German
Infection and Immunity	○	○	B<br>1998–2006<br>Print ver.	US<br>English
Infection Control and Hospital Epidemiology: The official journal of the Society of Hospital Epidemiologists of America	○	○	B<br>1998–2006<br>Print ver.	US<br>English
Infectious Disease Clinics of North America	○	○	B<br>1998–2006<br>Print ver.	US<br>English
Infectious Diseases in Obstetrics and Gynecology	○		B<br>1998–2006<br>Print ver.	Egypt<br>English

Infectious Disorders Drug Targets	○		D<br>2006<br>Print ver.	United Arb Emirates<br>English
International Journal of Antimicrobial Agents	○	○	D<br>1998–2006<br>Print ver.	Netherlands,<br>English
International Journal of Hygiene and Environmental Health	○	○	F<br>2000–2006<br>Print ver.	Germany<br>English
International Journal of Infectious Diseases: IJID: Official publication of the International Society for Infectious Diseases	○	○	B<br>1998–2006<br>Print ver.	Canada<br>English
International Journal of Medical Microbiology: IJMM (*Zentralblatt für Bakteriologie* before 2000)	○		C<br>1998–2006<br>Print ver.	Germany<br>English
International Journal of STD & AIDS	○	○	A&B<br>1998–2006<br>Print ver.	UK<br>English

The International Journal of Tuberculosis and Lung Disease: The official journal of the International Union against Tuberculosis and Lung Disease	○	○	A<br>1998–2006<br>Print ver.	France<br>English
Journal of Acquired Immune Deficiency Syndromes (1999)	○	○	A<br>1999–2006<br>Print ver.	US<br>English
The Japanese Journal of Antibiotics	○		D<br>1998–2006<br>Print ver.	Japan<br>Japanese<br>Asian journal
Japanese Journal of Infectious Diseases	○	○	B<br>1999–2006<br>Print ver.	Japan<br>English<br>Asian journal
Nihon Hansenbyō Gakkai zasshi = Japanese Journal of Leprosy: Official organ of the Japanese Leprosy Association	○		A<br>1998–2006<br>Print ver.	Japan<br>Japanese<br>English<br>Asian journal
Nihon Ishinkin Gakkai zasshi = Japanese Journal of Medical Mycology	○		C<br>1998–2006<br>Print ver.	Japan<br>Japanese<br>English<br>Asian journal

The Journal of Antibiotics	○		D<br>1998–2006<br>Print ver.	Japan<br>English<br>Asian journal
The Journal of Antimicrobial Chemotherapy	○	○	D<br>1998–2006<br>Print ver.	UK<br>English
Journal of Clinical Microbiology	○		C<br>1998–2006<br>Print ver.	US<br>English
Journal of Clinical Virology: The official publication of the Pan American Society for Clinical Virology	○		C<br>1998–2006<br>Print ver.	Netherlands<br>English
Journal of Communicable Diseases	○		B<br>1998–2006<br>Print ver.	India<br>English
The Journal of Hospital Infection	○	○	B<br>1998–2006<br>Print ver.	UK<br>English
Journal of Immune Based Therapies and Vaccines	○		D<br>2003–2006<br>Electronic ver.	UK<br>English
The Journal of Infection	○	○	B<br>1998–2006	UK<br>English

			Print ver.	
Journal of Infection and Chemotherapy: Official journal of the Japan Society of Chemotherapy	○		B&D<br>1998–2006<br>Print ver.	Japan<br>English<br>Asian journal
The Journal of Infectious Diseases	○	○	B<br>1998–2006<br>Print ver.	US<br>English
Journal of Medical Microbiology	○		C<br>1998–2006<br>Print ver.	UK<br>English
Journal of Medical Virology	○		C<br>1998–2006<br>Print ver.	US<br>English
Journal of Microbiological Methods	○		E<br>1998–2006<br>Print ver.	Netherlands<br>English
Journal of Microbiology, Immunology, and Infection = Wei mian yu gan ran za zhi	○		B<br>1998–2006<br>Print ver.	China<br>Chinese<br>English<br>Asian journal
Journal of Vector-borne Diseases	○		B<br>2003–2006<br>Print ver.	India<br>English<br>Asian journal

Journal of Viral Hepatitis	○	○	A<br>1998–2006<br>Print ver.	UK<br>English
Journal of Virological Methods	○		E<br>1998–2006<br>Print ver.	Netherlands<br>English
Kansenshogaku zasshi: The Journal of the Japanese Association for Infectious Diseases	○		B<br>1998–2006<br>Print ver.	Japan<br>Japanese<br>Asian journal
Kekkaku: [Tuberculosis]	○		A<br>1998–2006<br>Print ver.	Japan<br>Japanese<br>Asian journal
The Lancet Infectious Diseases	○	○	B<br>2001–2006<br>Print ver.	US<br>English
Leprosy Review	○	○	A<br>1998–2006<br>Print ver.	UK<br>English
Malaria Journal	○		A<br>2002–2006<br>Electronic ver.	UK<br>English
Médecine et Maladies Infectieuses	○	○	A<br>1998–2006<br>Print ver.	France<br>French

Medical Microbiology and Immunology	○		C<br>1998–2006<br>Print ver.	Germany<br>English
Medical Mycology: Official publication of the International Society for Human and Animal Mycology	○		C<br>1998–2006<br>Print ver.	UK<br>English
Microbes and Infection / Institut Pasteur	○		B<br>1999–2006<br>Print ver.	France<br>English
Microbial Drug Resistance (Larchmont, N.Y.)	○	○	D<br>1998–2006<br>Print ver.	US<br>English
Mycoses	○		A<br>1998–2006<br>Print ver.	Germany<br>English
The Pediatric Infectious Disease Journal	○	○	B<br>1998–2006<br>Print ver.	US<br>English
Problemy tuberkuleza i boleznei legkikh	○		A<br>2003-2006<br>Print ver.	Russia<br>Russian
Reviews in Medical	○		C	UK

Virology			1998–2006<br>Print ver.	English
Scandinavian Journal of Infectious Diseases	○	○	B<br>1998–2006<br>Print ver.	Sweden<br>English
Sexually Transmitted Diseases	○	○	B<br>1998–2006<br>Print ver.	US<br>English
Sexually Transmitted Infections	○	○	B<br>1998–2006<br>Print ver.	UK<br>English
Surgical Infections	○		B<br>2000–2006<br>Print ver.	US<br>English
The Brazilian Journal of Infectious Diseases: An official publication of the Brazilian Society of Infectious Diseases	○	○	B<br>1998–2006<br>Print ver.	Brazil<br>English
Transplant Infectious Disease: An official journal of the Transplantation Society	○	○	B<br>1999–2006<br>Print ver.	Denmark<br>English
Travel Medicine and Infectious Disease	○		B<br>2003–2006	Netherlands<br>English

			Print ver.	
Tropical Medicine & International Health : TM & IH	○		F<br>1998–2006<br>Print ver.	UK<br>English
Tuberculosis (Edinburgh, Scotland)	○		A<br>2001–2006<br>Print ver.	UK<br>English
Tuberkuloz ve toraks	○		A<br>2003-2006<br>Print ver.	Turkey<br>Turkish
Vaccine	○		D<br>1998–2006<br>Print ver.	Netherlands<br>English
Vector-borne and Zoonotic Diseases (Larchmont, N.Y.)	○	○	B<br>2001–2006<br>Print ver.	US<br>English
Zhonghua jie he he hu xi za zhi = Zhonghua jiehe he huxi zazhi = Chinese Journal of Tuberculosis and Respiratory Diseases	○		A<br>1998–2006<br>Print ver.	China<br>Chinese<br>Asian journal
Zhonghua shi yan he lin chuang bing du xue za zhi =	○		C<br>1998–2006<br>Print ver.	China<br>Chinese<br>Asian journal

Zhonghua shiyan he linchuang bingduxue zazhi = Chinese Journal of Experimental and Clinical Virology

---

[*]Journal titles based on PubMed database [34]

[†]Journals registered in the databases, which had been published on the Web in February 2008

[‡]Checked on January 27, 2009

[§]Shown in Figure 1

[¶]Information about languages was from the PubMed database [34] and the National Library of Medicine (NLM) Catalog [44] of the National Center for Biotechnology Information. In the case of including English and other languages, such as German, French, Japanese and Chinese, the former was the main language

[#]Journal titles were from the PubMed database [34]

[**]Journals marked in the column were equal to those registered in the 'Infectious Disease Category' of the Science Citation Index Expanded[TM], except for 8 journals (total 56 journals registered in the category on January 27, 2009). The 8 journals were: Canadian Journal of Infectious Diseases & Medical Microbiology, which ended publication in 2004, Infection Genetics and Evolution, which started in 2001, Infections in Medicine, for which almost no articles were registered in the PubMed database, Revista Chilena de Infectologia, articles from which have been registered in the PubMed database since 2005, Sexual Health, which started in 2004, the Southern African Journal of HIV Medicine, full text articles from which have been found since 2004 in the PubMed database, Transboundary and Emerging Diseases, and Zoonoses and Public Health, which started in 2008 and 2007, respectively.

[††]Journals which have print version only or both print & electronic versions

[‡‡]Journals which have an electronic version only
