# Supplementary material for: New journal selection for quantitative survey of infectious disease research: application for Asian trend analysis
Source: BMC Med Res Methodol. 2009 Oct 6;9:67. doi: 10.1186/1471-2288-9-67 (PMC2766390; doi:10.1186/1471-2288-9-67)
Supplement: Additional file 3 — Number of infectious disease research articles in the 100 journals. The data shows the number of infectious disease research articles published in the 100 newly selected journals. [file 1471-2288-9-67-S3.PDF]

Additional file 3 Number of infectious disease research articles in the 100 journals

| | 1998 OR* | 1998 RV† | 1999 OR | 1999 RV | 2000 OR | 2000 RV | 2001 OR | 2001 RV | 2002 OR | 2002 RV | 2003 OR | 2003 RV | 2004 OR | 2004 RV | 2005 OR | 2005 RV | 2006 OR | 2006 RV | 1998 ~2006 OR | 1998 ~2006 RV | 1998 ~2006 OR+RV |
|---|---|---|---|---|---|---|---|---|---|---|---|---|---|---|---|---|---|---|---|---|---|
| Japan (number of articles) | 573 | 33 | 684 | 46 | 736 | 43 | 670 | 55 | 733 | 42 | 704 | 65 | 694 | 56 | 696 | 47 | 712 | 57 | 6202 | 444 | 6646 |
| Relative to Asian countries (%) | 69.0 | 68.8 | 63.4 | 85.2 | 55.3 | 71.7 | 48.2 | 74.3 | 48.4 | 63.6 | 43.9 | 60.7 | 38.0 | 58.3 | 37.7 | 47.5 | 35.4 | 54.3 | 46.1 | 63.0 | 46.9 |
| Relative to the world (%) | 5.9 | 3.3 | 6.6 | 3.7 | 6.8 | 3.0 | 6.1 | 3.7 | 6.6 | 3.0 | 5.8 | 4.1 | 5.4 | 3.5 | 5.3 | 3.0 | 5.0 | 3.7 | 5.9 | 3.5 | 5.6 |
| China (number of articles) | 22 | 1 | 41 | 0 | 224 | 0 | 261 | 3 | 255 | 1 | 272 | 4 | 350 | 9 | 371 | 10 | 397 | 6 | 2194 | 33 | 2227 |
| Relative to Asian countries (%) | 2.7 | 2.1 | 3.8 | 0 | 16.8 | 0 | 18.8 | 4.1 | 16.8 | 1.5 | 16.9 | 3.7 | 19.2 | 9.4 | 20.1 | 10.1 | 19.8 | 5.7 | 16.3 | 4.7 | 15.7 |
| Relative to the world (%) | 0.2 | 0.1 | 0.4 | 0 | 2.1 | 0 | 2.4 | 0.2 | 2.3 | 0.1 | 2.2 | 0.3 | 2.7 | 0.6 | 2.8 | 0.6 | 2.8 | 0.4 | 2.1 | 0.3 | 1.9 |
| Korea (number of articles) | 26 | 1 | 45 | 1 | 58 | 2 | 82 | 2 | 69 | 2 | 118 | 4 | 118 | 2 | 127 | 2 | 152 | 3 | 796 | 19 | 814 |
| Relative to Asian countries (%) | 3.1 | 2.1 | 4.2 | 1.9 | 4.4 | 3.3 | 5.9 | 2.7 | 4.6 | 3.0 | 7.4 | 3.7 | 6.5 | 2.1 | 6.9 | 2.0 | 7.6 | 2.9 | 5.9 | 2.7 | 5.8 |
| Relative to the world (%) | 0.3 | 0.1 | 0.4 | 0.1 | 0.5 | 0.1 | 0.8 | 0.1 | 0.6 | 0.1 | 1.0 | 0.3 | 0.9 | 0.1 | 1.0 | 0.1 | 1.1 | 0.2 | 0.8 | 0.1 | 0.7 |
| India (number of articles) | 116 | 6 | 138 | 5 | 138 | 9 | 171 | 4 | 238 | 8 | 293 | 19 | 342 | 5 | 343 | 19 | 401 | 20 | 2188 | 95 | 2283 |
| Relative to Asian countries (%) | 14.0 | 12.5 | 12.8 | 9.3 | 10.4 | 15.0 | 12.3 | 5.4 | 15.7 | 12.1 | 18.3 | 17.8 | 18.7 | 5.2 | 18.6 | 19.2 | 20.0 | 19.0 | 16.3 | 13.5 | 16.1 |
| Relative to the world (%) | 1.2 | 0.6 | 1.3 | 0.4 | 1.3 | 0.6 | 1.6 | 0.3 | 2.2 | 0.6 | 2.4 | 1.2 | 2.7 | 0.3 | 2.6 | 1.2 | 2.8 | 1.3 | 2.1 | 0.7 | 1.9 |
| Thailand (number of articles) | 1 | 0 | 3 | 0 | 7 | 0 | 7 | 1 | 10 | 1 | 11 | 1 | 6 | 1 | 12 | 2 | 15 | 0 | 72 | 6 | 78 |
| Relative to Asian countries (%) | 0.1 | 0 | 0.3 | 0 | 0.5 | 0 | 0.5 | 1.4 | 0.7 | 1.5 | 0.7 | 0.9 | 0.3 | 1.0 | 0.6 | 2.0 | 0.7 | 0 | 0.5 | 0.9 | 0.6 |
| Relative to the world (%) | 0.1> | 0 | 0.1> | 0 | 0.1 | 0 | 0.1 | 0.1 | 0.1 | 0.1 | 0.1 | 0.1 | 0.1> | 0.1 | 0.1 | 0.1 | 0.1 | 0 | 0.1 | 0.1> | 0.1 |
| Indonesia (number of articles) | 7 | 0 | 15 | 0 | 10 | 1 | 18 | 0 | 22 | 0 | 17 | 0 | 6 | 1 | 12 | 0 | 8 | 0 | 115 | 2 | 117 |
| Relative to Asian countries (%) | 0.8 | 0 | 1.4 | 0 | 0.8 | 1.7 | 1.3 | 0 | 1.5 | 0 | 1.1 | 0 | 0.3 | 1.0 | 0.6 | 0 | 0.4 | 0 | 0.9 | 0.3 | 0.8 |
| Relative to the world (%) | 0.1 | 0 | 0.1 | 0 | 0.1 | 0.1 | 0.2 | 0 | 0.2 | 0 | 0.1 | 0 | 0.1> | 0.1 | 0.1 | 0 | 0.1 | 0 | 0.1 | 0.1> | 0.1 |
| Taiwan (number of articles) | 62 | 5 | 99 | 1 | 110 | 3 | 140 | 7 | 140 | 8 | 124 | 7 | 210 | 11 | 204 | 12 | 244 | 12 | 1333 | 66 | 1399 |
| Relative to Asian countries (%) | 7.5 | 10.4 | 9.2 | 1.9 | 8.3 | 5.0 | 10.1 | 9.5 | 9.2 | 12.1 | 7.7 | 6.5 | 11.5 | 11.5 | 11.0 | 12.1 | 12.1 | 11.4 | 9.9 | 9.4 | 9.9 |
| Relative to the world (%) | 0.6 | 0.5 | 0.9 | 0.1 | 1.0 | 0.2 | 1.3 | 0.5 | 1.3 | 0.6 | 1.0 | 0.4 | 1.6 | 0.7 | 1.5 | 0.8 | 1.7 | 0.8 | 1.3 | 0.5 | 1.2 |
| Singapore (number of articles) | 8 | 2 | 16 | 0 | 16 | 0 | 20 | 0 | 29 | 3 | 31 | 1 | 58 | 5 | 40 | 7 | 35 | 7 | 253 | 25 | 278 |
| Relative to Asian countries (%) | 1.0 | 4.2 | 1.5 | 0 | 1.2 | 0 | 1.4 | 0 | 1.9 | 4.5 | 1.9 | 0.9 | 3.2 | 5.2 | 2.2 | 7.1 | 1.7 | 6.7 | 1.9 | 3.5 | 2.0 |
| Relative to the world (%) | 0.1 | 0.2 | 0.2 | 0 | 0.1 | 0 | 0.2 | 0 | 0.3 | 0.2 | 0.3 | 0.1 | 0.4 | 0.3 | 0.3 | 0.4 | 0.2 | 0.5 | 0.2 | 0.2 | 0.2 |

| | | | | | | | | | | | | | | | | | | | | | |
|---|---|---|---|---|---|---|---|---|---|---|---|---|---|---|---|---|---|---|---|---|---|
| Malaysia (number of articles) | 10 | 0 | 13 | 0 | 14 | 1 | 11 | 0 | 10 | 0 | 15 | 3 | 16 | 4 | 20 | 0 | 21 | 0 | 131 | 7 | 138 |
| Relative to Asian countries (%) | 1.2 | 0 | 1.2 | 0 | 1.1 | 1.7 | 0.8 | 0 | 0.7 | 0 | 0.9 | 2.8 | 0.9 | 4.2 | 1.1 | 0 | 1.0 | 0 | 1.0 | 1.0 | 1.0 |
| Relative to the world (%) | 0.1 | 0 | 0.1 | 0 | 0.1 | 0.1 | 0.1 | 0 | 0.1 | 0 | 0.1 | 0.2 | 0.1 | 0.3 | 0.2 | 0 | 0.1 | 0 | 0.1 | 0.1 | 0.1 |
| Philippines (number of articles) | 5 | 0 | 12 | 0 | 10 | 0 | 3 | 1 | 3 | 0 | 11 | 1 | 7 | 1 | 7 | 0 | 4 | 0 | 61 | 4 | 65 |
| Relative to Asian countries (%) | 0.6 | 0 | 1.1 | 0 | 0.8 | 0 | 0.2 | 1.4 | 0.2 | 0 | 0.7 | 0.9 | 0.4 | 1.0 | 0.4 | 0 | 0.2 | 0 | 0.5 | 0.6 | 0.5 |
| Relative to the world (%) | 0.1 | 0 | 0.1 | 0 | 0.1 | 0 | 0.1> | 0.1 | 0.1> | 0 | 0.1 | 0.1 | 0.1 | 0.1 | 0.1 | 0 | 0.1> | 0 | 0.1 | 0.1> | 0.1 |
| Vietnam (number of articles) | 0 | 0 | 13 | 1 | 7 | 1 | 6 | 1 | 7 | 1 | 9 | 2 | 18 | 1 | 15 | 0 | 21 | 0 | 107 | 4 | 111 |
| Relative to Asian countries (%) | 0 | 0 | 1.2 | 1.9 | 0.5 | 1.7 | 0.4 | 1.4 | 0.5 | 1.5 | 0.6 | 1.9 | 1.0 | 1.0 | 0.8 | 0 | 1.0 | 0 | 0.8 | 0.6 | 0.8 |
| Relative to the world (%) | 0 | 0 | 0.1 | 0.1 | 0.1 | 0.1 | 0.1 | 0.1 | 0.1 | 0.1 | 0.1 | 0.1 | 0.1 | 0.1 | 0.1 | 0 | 0.1 | 0 | 0.1 | 0.1> | 0.1 |
| total number of articles in Asian countries | 830 | 48 | 1079 | 54 | 1330 | 60 | 1389 | 74 | 1516 | 66 | 1605 | 107 | 1825 | 96 | 1847 | 99 | 2010 | 105 | 13452 | 705 | 14156 |
| Relative to the world (%) | 8.6 | 4.7 | 10.3 | 4.4 | 12.4 | 4.2 | 12.7 | 5.0 | 13.7 | 4.7 | 13.1 | 6.8 | 14.2 | 6.1 | 13.9 | 6.3 | 14.2 | 6.9 | 12.8 | 5.5 | 12.0 |
| total number of articles in the world | 9661 | 1013 | 10430 | 1237 | 10764 | 1442 | 10919 | 1484 | 11032 | 1405 | 12239 | 1584 | 12896 | 1580 | 13246 | 1580 | 14121 | 1525 | 105308 | 12850 | 118158 |

*Original articles
†Reviews
