# Supplementary material for: New journal selection for quantitative survey of infectious disease research: application for Asian trend analysis
Source: BMC Med Res Methodol. 2009 Oct 6;9:67. doi: 10.1186/1471-2288-9-67 (PMC2766390; doi:10.1186/1471-2288-9-67)
Supplement: Additional file 4 — Number of infectious disease research articles in the SCI Infectious Disease Category. The data shows the number of infectious disease research articles published in the journals registered in the 'Infectious Disease Category' of the Science Citation Index Expanded™. [file 1471-2288-9-67-S4.PDF]

Additional file 4 Number of infectious disease research articles in the SCI Infectious Disease Category

SCI infectious disease category	1998 OR*	1998 RV†	1999 OR	1999 RV	2000 OR	2000 RV	2001 OR	2001 RV	2002 OR	2002 RV	2003 OR	2003 RV	2004 OR	2004 RV	2005 OR	2005 RV	2006 OR	2006 RV	1998 ~2006 OR	1998 ~2006 RV	1998 ~2006 OR+RV
Japan (number of articles)	143	2	210	12	263	11	240	20	234	12	219	10	216	12	224	9	231	7	1980	95	2075
Relative to Asian countries (%)	55.2	28.6	57.5	85.7	60.0	55.0	53.3	80.0	49.6	42.9	45.6	38.5	35.0	31.6	35.6	27.3	33.1	33.0	44.9	45.5	44.9
Relative to the world (%)	2.6	0.3	3.6	1.5	4.2	1.2	3.8	2.0	3.7	1.4	3.3	1.1	3.1	1.2	3.1	0.9	3.1	0.8	3.4	1.2	3.1
China (number of articles)	3	1	11	0	15	0	22	1	27	0	35	2	70	8	68	7	78	2	329	21	350
Relative to Asian countries (%)	1.2	14.3	3.0	0	3.4	0	4.9	4.0	5.7	0	7.3	7.7	11.3	21.1	10.8	21.2	11.2	9.5	7.5	10.0	7.6
Relative to the world (%)	0.1	0.1	0.2	0	0.2	0	0.4	0.1	0.4	0	0.5	0.2	1.0	0.8	0.9	0.7	1.0	0.2	0.6	0.3	0.5
Korea (number of articles)	10	0	16	0	20	0	41	0	31	1	41	3	57	0	67	1	83	1	367	6	373
Relative to Asian countries (%)	3.9	0	4.4	0	4.6	0	9.1	0	6.6	3.6	8.5	11.5	9.2	0	10.7	3.0	11.9	4.8	8.3	2.9	8.1
Relative to the world (%)	0.2	0	0.3	0	0.3	0	0.7	0	0.5	0.1	0.6	0.3	0.8	0	0.9	0.1>	1.1	0.1	0.6	0.1	0.6
India (number of articles)	51	3	67	2	72	6	63	1	91	4	96	4	128	2	129	7	138	3	835	32	867
Relative to Asian countries (%)	19.7	42.9	18.4	14.3	16.4	30.0	14.0	4.0	19.3	14.3	20.0	15.4	20.7	5.3	20.5	21.2	19.8	14.3	18.9	15.3	18.8
Relative to the world (%)	0.9	0.4	1.1	0.2	1.2	0.7	1.0	0.1	1.5	0.5	1.5	0.4	1.8	0.2	1.8	0.7	1.8	0.3	1.4	0.4	1.3
Thailand (number of articles)	1	0	2	0	5	0	4	1	7	1	7	1	6	1	11	1	10	0	53	5	58
Relative to Asian countries (%)	0.4	0	0.5	0	1.1	0	0.9	4.0	1.5	3.6	1.5	3.8	1.0	2.6	1.7	3.0	1.4	0	1.2	2.4	1.3
Relative to the world (%)	0.1>	0	0.1>	0	0.1	0	0.1	0.1	0.1	0.1	0.1	0.1	0.1	0.1	0.2	0.1	0.1	0	0.1	0.1	0.1
Indonesia (number of articles)	5	0	3	0	5	1	4	0	6	0	6	0	4	0	4	0	3	0	40	1	41
Relative to Asian countries (%)	1.9	0	0.8	0	1.1	5.0	0.9	0	1.3	0	1.3	0	0.6	0	0.6	0	0.4	0	0.9	0.5	0.9
Relative to the world (%)	0.1	0	0.1	0	0.1	0.1	0.1	0	0.1	0	0.1	0	0.1	0	0.1	0	0.1>	0	0.1	0.1>	0.1
Taiwan (number of articles)	27	0	26	0	31	1	52	1	52	6	40	1	81	9	82	4	117	4	508	26	534
Relative to Asian countries (%)	10.4	0	7.1	0	7.1	5.0	11.6	4.0	11.0	21.4	8.3	3.8	13.1	23.7	13.0	12.1	16.8	19.0	11.5	12.4	11.6
Relative to the world (%)	0.5	0	0.4	0	0.5	0.1	0.8	0.1	0.8	0.7	0.6	0.1	1.2	0.9	1.1	0.4	1.6	0.5	0.9	0.3	0.8
Singapore (number of articles)	5	1	9	0	8	0	9	0	14	3	17	1	34	5	25	4	19	4	140	18	158
Relative to Asian countries (%)	1.9	14.3	2.5	0	1.8	0	2.0	0	3.0	10.7	3.5	3.8	5.5	13.2	4.0	12.1	2.7	19.0	3.2	8.6	3.4

Relative to the world (%)	0.1	0.1	0.2	0	0.1	0	0.1	0	0.2	0.3	0.3	0.1	0.5	0.5	0.3	0.4	0.3	0.5	0.2	0.2	0.2
Malaysia (number of articles)	7	0	6	0	9	0	8	0	3	0	6	1	9	0	10	0	6	0	64	1	65
Relative to Asian countries (%)	2.7	0	1.6	0	2.1	0	1.8	0	0.6	0	1.3	3.8	1.5	0	1.6	0	0.9	0	1.5	0.5	1.4
Relative to the world (%)	0.1	0	0.1	0	0.1	0	0.1	0	0.1>	0	0.1	0.1	0.1	0	0.1	0	0.1	0	0.1	0.1>	0.1
Philippines (number of articles)	2	0	6	0	8	0	3	0	2	0	6	1	3	0	1	0	1	0	32	1	33
Relative to Asian countries (%)	0.8	0	1.6	0	1.8	0	0.7	0	0.4	0	1.3	3.8	0.5	0	0.2	0	0.1	0	0.7	0.5	0.7
Relative to the world (%)	0.1>	0	0.1	0	0.1	0	0.1>	0	0.1>	0	0.1	0.1	0.1>	0	0.1>	0	0.1>	0	0.1	0.1>	0.1>
Vietnam (number of articles)	5	0	9	0	2	1	4	1	5	1	7	2	9	1	8	0	12	0	64	3	67
Relative to Asian countries (%)	1.9	0	2.5	0	0.5	5.0	0.9	4.0	1.1	3.6	1.5	7.7	1.5	2.6	1.3	0	1.7	0	1.5	1.4	1.4
Relative to the world (%)	0.1	0	0.2	0	0.1>	0.1	0.1	0.1	0.1	0.1	0.1	0.2	0.1	0.1	0.1	0	0.2	0	0.1	0.1>	0.1
total number of articles in Asian countries	259	7	365	14	438	20	450	25	472	28	480	26	617	38	629	33	698	21	4412	209	4621
Relative to the world (%)	4.7	1.0	6.2	1.7	7.0	2.2	7.2	2.6	7.6	3.2	7.3	2.8	8.8	3.9	8.8	3.2	9.3	2.4	7.6	2.6	6.9
total number of articles in the world	5489	697	5847	805	6256	919	6262	976	6243	864	6615	932	7039	981	7163	1043	7510	877	58424	8094	66518

*Original articles
†Reviews
